# 1-(4-Bromo­phenyl­sulfin­yl)-2-methyl­naphtho­[2,1-*b*]furan

**DOI:** 10.1107/S1600536812012603

**Published:** 2012-03-31

**Authors:** Hong Dae Choi, Pil Ja Seo, Uk Lee

**Affiliations:** a Department of Chemistry, Dongeui University, San 24 Kaya-dong Busanjin-gu, Busan 614-714, Republic of Korea; b Department of Chemistry, Pukyong National University, 599-1 Daeyeon 3-dong, Nam-gu, Busan 608-737, Republic of Korea

## Abstract

In the title compound, C_19_H_13_BrO_2_S, the 4-bromo­phenyl ring makes a dihedral angle of 83.75 (4)° with the mean plane of the naphtho­furan fragment [r.m.s. deviation = 0.024 (2) Å]. In the crystal, mol­ecules are linked *via* pairs of C—H⋯O hydrogen bonds, forming inversion dimers. These dimers are connected by weak π–π inter­actions between the central naphtho­furan benzene rings of neighbouring mol­ecules [centroid–centroid distance = 3.483 (2) Å, inter­planar distance = 3.416 (2) Å and slippage = 0.680 (2) Å].

## Related literature

For background information and the crystal structures of related compounds, see: Choi *et al.* (2007 ▶, 2012 ▶).
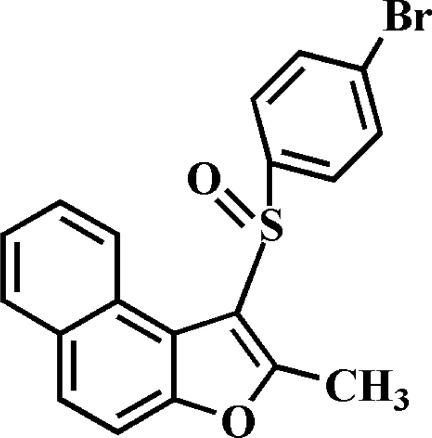


## Experimental

### 

#### Crystal data

C_19_H_13_BrO_2_S
*M* *_r_* = 385.26
Triclinic, 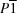

*a* = 8.7124 (1) Å
*b* = 9.4857 (1) Å
*c* = 10.2898 (1) Å
α = 82.883 (1)°
β = 71.126 (1)°
γ = 75.672 (1)°
*V* = 778.74 (2) Å^3^
*Z* = 2
Mo *K*α radiation
μ = 2.78 mm^−1^
*T* = 173 K
0.33 × 0.29 × 0.27 mm

#### Data collection

Bruker SMART APEXII CCD diffractometer
Absorption correction: multi-scan (*SADABS*; Bruker, 2009 ▶) *T*_min_ = 0.462, *T*_max_ = 0.517
14577 measured reflections
3853 independent reflections
3461 reflections with *I* > 2σ(*I*)
*R*_int_ = 0.031

#### Refinement

*R*[*F* ^2^ > 2σ(*F* ^2^)] = 0.030
*wR*(*F* ^2^) = 0.080
*S* = 1.05
3853 reflections
209 parameters
H-atom parameters constrained
Δρ_max_ = 0.62 e Å^−3^
Δρ_min_ = −0.60 e Å^−3^

### 

Data collection: *APEX2* (Bruker, 2009 ▶); cell refinement: *SAINT* (Bruker, 2009 ▶); data reduction: *SAINT*; program(s) used to solve structure: *SHELXS97* (Sheldrick, 2008 ▶); program(s) used to refine structure: *SHELXL97* (Sheldrick, 2008 ▶); molecular graphics: *ORTEP-3* (Farrugia, 1997 ▶) and *DIAMOND* (Brandenburg, 1998 ▶); software used to prepare material for publication: *SHELXL97*.

## Supplementary Material

Crystal structure: contains datablock(s) global, I. DOI: 10.1107/S1600536812012603/sj5222sup1.cif

Structure factors: contains datablock(s) I. DOI: 10.1107/S1600536812012603/sj5222Isup2.hkl

Supplementary material file. DOI: 10.1107/S1600536812012603/sj5222Isup3.cml

Additional supplementary materials:  crystallographic information; 3D view; checkCIF report

## Figures and Tables

**Table 1:** Hydrogen-bond geometry (Å, °)

*D*—H⋯*A*	*D*—H	H⋯*A*	*D*⋯*A*	*D*—H⋯*A*
C19—H19⋯O2^i^	0.95	2.35	3.221 (2)	152

Symmetry code: (i) 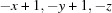.

**Fig. 1.**
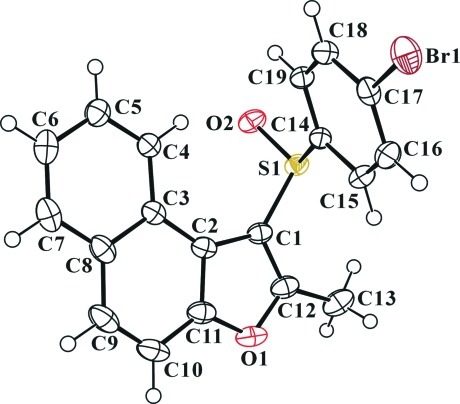
The molecular structure of the title compound with the atom numbering scheme. Displacement ellipsoids are drawn at the 50% probability level. H atoms are presented as small spheres of arbitrary radius.

**Fig. 2.**
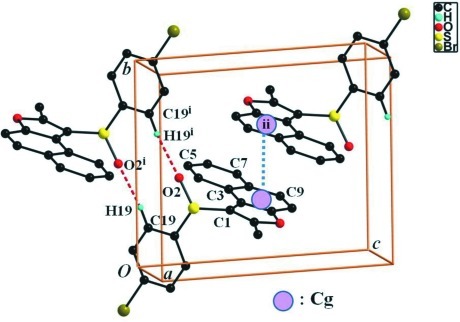
A view of the C—H···O and π–π interactions (dotted lines) in the crystal structure of the title compound. H atoms non-participating in hydrogen-bonding were omitted for clarity. [Symmetry codes: (i) - x + 1, - y + 1, - z ; (ii) - x + 1 , - y + 1, - z + 1.]

## supplementary crystallographic information

### Comment

As a part of our ongoing study of 2-methylnaphtho[2,1-b]furan derivatives containing 1-phenylsulfinyl (Choi *et al.*, 2007) and 1-(4-methylphenylsulfinyl) (Choi *et al.*, 2012) substituents, we report herein the crystal structure of the title compound.

In the title molecule (Fig. 1), the naphthofuran unit is essentially planar, with a mean deviation of 0.024 (2) Å from the least-squares plane defined by the thirteen constituent atoms. The dihedral angle between the 4-bromophenyl ring and the mean plane of the naphthofuran fragment is 83.75 (4)°. In the crystal structure, molecules are linked via pairs of C—H···O hydrogen bonds (Fig. 2 & Table 1), forming inversion dimers. These dimers are connected by weak π–π interactions between the central naphthofuran benzene rings of neighbouring molecules, with a Cg···Cg^ii^ distance of 3.483 (2) Å and an interplanar distance of 3.416 (2) Å resulting in a slippage of 0.680 (2) Å (Fig. 2, Cg is the centroid of the C2/C3/C8/C9/C10/C11 benzene ring).

### Experimental

77% 3-Chloroperoxybenzoic acid (202 mg, 0.9 mmol) was added in small portions to a stirred solution of 1-(4-bromophenylsulfanyl)-2-methylnaphtho [2,1-b]furan (295 mg, 0.8 mmol) in dichloromethane (40 mL) at 273 K. After being stirred at room temperature for 5h, the mixture was washed with saturated sodium bicarbonate solution and the organic layer was separated, dried over magnesium sulfate, filtered and concentrated at reduced pressure. The residue was purified by column chromatography (hexane–ethyl acetate, 2:1 v/v) to afford the title compound as a colorless solid [yield 74%, m.p. 466–467 K; *R*_f_ = 0.59 (hexane–ethyl acetate, 2:1 v/v)]. Single crystals suitable for X-ray diffraction were prepared by slow evaporation of a solution of the title compound in acetone at room temperature.

### Refinement

All H atoms were positioned geometrically and refined using a riding model, with C—H = 0.95 Å for aryl and 0.98 Å for the methyl H atoms. Uiso(H) = 1.2*U*_eq_(C) for aryl and 1.5*U*_eq_(C) for methyl H atoms. The positions of the methyl H atoms were optimized rotationally.

### Crystal data

**Table:**

C_19_H_13_BrO_2_S	*Z* = 2
*M**_r_* = 385.26	*F*(000) = 388
Triclinic, *P*1	*D*_x_ = 1.643 Mg m^−^^3^
Hall symbol: -P 1	Mo *K*α radiation, λ = 0.71073 Å
*a* = 8.7124 (1) Å	Cell parameters from 7454 reflections
*b* = 9.4857 (1) Å	θ = 2.5–28.3°
*c* = 10.2898 (1) Å	µ = 2.78 mm^−^^1^
α = 82.883 (1)°	*T* = 173 K
β = 71.126 (1)°	Block, colourless
γ = 75.672 (1)°	0.33 × 0.29 × 0.27 mm
*V* = 778.74 (2) Å^3^	

### Data collection

**Table:**

Bruker SMART APEXII CCD diffractometer	3853 independent reflections
Radiation source: rotating anode	3461 reflections with *I* > 2σ(*I*)
Graphite multilayer monochromator	*R*_int_ = 0.031
Detector resolution: 10.0 pixels mm^-1^	θ_max_ = 28.3°, θ_min_ = 2.1°
φ and ω scans	*h* = −11→11
Absorption correction: multi-scan (*SADABS*; Bruker, 2009)	*k* = −12→12
*T*_min_ = 0.462, *T*_max_ = 0.517	*l* = −13→13
14577 measured reflections	

### Refinement

**Table:**

Refinement on *F*^2^	Primary atom site location: structure-invariant direct methods
Least-squares matrix: full	Secondary atom site location: difference Fourier map
*R*[*F*^2^ > 2σ(*F*^2^)] = 0.030	Hydrogen site location: difference Fourier map
*wR*(*F*^2^) = 0.080	H-atom parameters constrained
*S* = 1.05	*w* = 1/[σ^2^(*F*_o_^2^) + (0.0424*P*)^2^ + 0.3115*P*] where *P* = (*F*_o_^2^ + 2*F*_c_^2^)/3
3853 reflections	(Δ/σ)_max_ = 0.002
209 parameters	Δρ_max_ = 0.62 e Å^−^^3^
0 restraints	Δρ_min_ = −0.60 e Å^−^^3^

### Special details

**Table:**

Geometry. All esds (except the esd in the dihedral angle between two l.s. planes) are estimated using the full covariance matrix. The cell esds are taken into account individually in the estimation of esds in distances, angles and torsion angles; correlations between esds in cell parameters are only used when they are defined by crystal symmetry. An approximate (isotropic) treatment of cell esds is used for estimating esds involving l.s. planes.
Refinement. Refinement of F^2^ against ALL reflections. The weighted R-factor wR and goodness of fit S are based on F^2^, conventional R-factors R are based on F, with F set to zero for negative F^2^. The threshold expression of F^2^ > 2sigma(F^2^) is used only for calculating R-factors(gt) etc. and is not relevant to the choice of reflections for refinement. R-factors based on F^2^ are statistically about twice as large as those based on F, and R- factors based on ALL data will be even larger.

### Fractional atomic coordinates and isotropic or equivalent isotropic displacement parameters (Å^2^)

**Table:**

	*x*	*y*	*z*	*U*_iso_*/*U*_eq_	
Br1	1.00152 (2)	−0.12635 (2)	−0.16983 (2)	0.03951 (8)	
S1	0.35059 (5)	0.29100 (5)	0.20858 (4)	0.02531 (10)	
O1	0.39380 (18)	0.19036 (15)	0.57662 (13)	0.0339 (3)	
O2	0.34216 (16)	0.44277 (15)	0.14815 (14)	0.0308 (3)	
C1	0.4076 (2)	0.27335 (19)	0.36023 (17)	0.0248 (3)	
C2	0.5387 (2)	0.31553 (19)	0.39413 (17)	0.0241 (3)	
C3	0.6643 (2)	0.39490 (19)	0.32814 (17)	0.0236 (3)	
C4	0.6876 (2)	0.46220 (19)	0.19438 (17)	0.0243 (3)	
H4	0.6154	0.4567	0.1439	0.029*	
C5	0.8127 (2)	0.5349 (2)	0.1373 (2)	0.0295 (4)	
H5	0.8265	0.5795	0.0475	0.035*	
C6	0.9214 (2)	0.5446 (2)	0.2102 (2)	0.0357 (4)	
H6	1.0098	0.5933	0.1689	0.043*	
C7	0.8993 (3)	0.4836 (2)	0.3402 (2)	0.0357 (4)	
H7	0.9721	0.4918	0.3892	0.043*	
C8	0.7706 (2)	0.4086 (2)	0.40403 (19)	0.0295 (4)	
C9	0.7452 (3)	0.3495 (2)	0.5420 (2)	0.0357 (4)	
H9	0.8165	0.3611	0.5910	0.043*	
C10	0.6220 (3)	0.2772 (2)	0.60542 (19)	0.0353 (4)	
H10	0.6045	0.2392	0.6976	0.042*	
C11	0.5225 (2)	0.2618 (2)	0.52789 (18)	0.0293 (4)	
C12	0.3248 (2)	0.2003 (2)	0.47280 (19)	0.0301 (4)	
C13	0.1834 (3)	0.1278 (3)	0.5032 (2)	0.0419 (5)	
H13A	0.1309	0.1541	0.4299	0.063*	
H13B	0.1017	0.1594	0.5912	0.063*	
H13C	0.2243	0.0219	0.5087	0.063*	
C14	0.5374 (2)	0.17880 (19)	0.10434 (17)	0.0234 (3)	
C15	0.5817 (3)	0.0335 (2)	0.14588 (19)	0.0313 (4)	
H15	0.5173	−0.0031	0.2308	0.038*	
C16	0.7191 (3)	−0.0578 (2)	0.0640 (2)	0.0333 (4)	
H16	0.7497	−0.1574	0.0916	0.040*	
C17	0.8116 (2)	−0.0017 (2)	−0.05890 (19)	0.0283 (4)	
C18	0.7683 (2)	0.1426 (2)	−0.10104 (18)	0.0286 (4)	
H18	0.8335	0.1792	−0.1855	0.034*	
C19	0.6289 (2)	0.2341 (2)	−0.01936 (17)	0.0259 (3)	
H19	0.5970	0.3332	−0.0480	0.031*	

### Atomic displacement parameters (Å^2^)

**Table:**

	*U*^11^	*U*^22^	*U*^33^	*U*^12^	*U*^13^	*U*^23^
Br1	0.03626 (13)	0.03675 (14)	0.04460 (13)	−0.00190 (9)	−0.01114 (9)	−0.01442 (9)
S1	0.0229 (2)	0.0272 (2)	0.0268 (2)	−0.00701 (17)	−0.01025 (16)	0.00538 (16)
O1	0.0371 (7)	0.0337 (8)	0.0231 (6)	−0.0025 (6)	−0.0056 (5)	0.0069 (5)
O2	0.0278 (6)	0.0283 (7)	0.0344 (7)	−0.0035 (5)	−0.0129 (5)	0.0092 (5)
C1	0.0234 (8)	0.0242 (9)	0.0231 (8)	−0.0015 (7)	−0.0060 (6)	0.0016 (6)
C2	0.0258 (8)	0.0217 (8)	0.0212 (7)	0.0024 (6)	−0.0078 (6)	−0.0021 (6)
C3	0.0239 (8)	0.0210 (8)	0.0239 (8)	0.0013 (6)	−0.0079 (6)	−0.0047 (6)
C4	0.0247 (8)	0.0214 (8)	0.0261 (8)	−0.0015 (7)	−0.0089 (6)	−0.0025 (6)
C5	0.0287 (9)	0.0254 (9)	0.0324 (9)	−0.0046 (7)	−0.0069 (7)	−0.0034 (7)
C6	0.0304 (9)	0.0314 (11)	0.0481 (11)	−0.0095 (8)	−0.0112 (8)	−0.0084 (8)
C7	0.0330 (10)	0.0342 (11)	0.0458 (11)	−0.0035 (8)	−0.0194 (9)	−0.0117 (8)
C8	0.0307 (9)	0.0272 (10)	0.0318 (9)	0.0023 (7)	−0.0147 (7)	−0.0092 (7)
C9	0.0418 (11)	0.0343 (11)	0.0346 (10)	0.0050 (8)	−0.0240 (9)	−0.0080 (8)
C10	0.0452 (11)	0.0334 (11)	0.0232 (8)	0.0054 (9)	−0.0152 (8)	−0.0022 (7)
C11	0.0313 (9)	0.0271 (9)	0.0240 (8)	0.0020 (7)	−0.0077 (7)	−0.0001 (7)
C12	0.0284 (9)	0.0273 (10)	0.0272 (8)	−0.0004 (7)	−0.0040 (7)	0.0026 (7)
C13	0.0353 (11)	0.0409 (12)	0.0419 (11)	−0.0124 (9)	−0.0024 (9)	0.0092 (9)
C14	0.0276 (8)	0.0228 (9)	0.0229 (8)	−0.0076 (7)	−0.0114 (6)	0.0020 (6)
C15	0.0411 (10)	0.0259 (10)	0.0266 (8)	−0.0120 (8)	−0.0092 (8)	0.0058 (7)
C16	0.0460 (11)	0.0202 (9)	0.0339 (9)	−0.0064 (8)	−0.0143 (8)	0.0017 (7)
C17	0.0314 (9)	0.0274 (9)	0.0300 (9)	−0.0063 (7)	−0.0132 (7)	−0.0061 (7)
C18	0.0326 (9)	0.0314 (10)	0.0236 (8)	−0.0103 (8)	−0.0096 (7)	0.0018 (7)
C19	0.0325 (9)	0.0229 (9)	0.0245 (8)	−0.0073 (7)	−0.0130 (7)	0.0044 (6)

### Geometric parameters (Å, º)

**Table:**

Br1—C17	1.8932 (19)	C8—C9	1.428 (3)
S1—O2	1.4899 (13)	C9—C10	1.360 (3)
S1—C1	1.7621 (17)	C9—H9	0.9500
S1—C14	1.7966 (19)	C10—C11	1.395 (3)
O1—C12	1.369 (2)	C10—H10	0.9500
O1—C11	1.378 (2)	C12—C13	1.486 (3)
C1—C12	1.360 (2)	C13—H13A	0.9800
C1—C2	1.451 (2)	C13—H13B	0.9800
C2—C11	1.382 (2)	C13—H13C	0.9800
C2—C3	1.424 (2)	C14—C19	1.384 (2)
C3—C4	1.417 (2)	C14—C15	1.389 (3)
C3—C8	1.427 (2)	C15—C16	1.381 (3)
C4—C5	1.366 (2)	C15—H15	0.9500
C4—H4	0.9500	C16—C17	1.385 (3)
C5—C6	1.411 (3)	C16—H16	0.9500
C5—H5	0.9500	C17—C18	1.380 (3)
C6—C7	1.363 (3)	C18—C19	1.390 (3)
C6—H6	0.9500	C18—H18	0.9500
C7—C8	1.413 (3)	C19—H19	0.9500
C7—H7	0.9500		
			
O2—S1—C1	110.36 (8)	C9—C10—H10	121.8
O2—S1—C14	107.32 (8)	C11—C10—H10	121.8
C1—S1—C14	97.62 (8)	O1—C11—C2	111.20 (16)
C12—O1—C11	106.47 (13)	O1—C11—C10	123.59 (16)
C12—C1—C2	107.38 (15)	C2—C11—C10	125.21 (18)
C12—C1—S1	119.35 (14)	C1—C12—O1	110.59 (16)
C2—C1—S1	133.18 (13)	C1—C12—C13	133.92 (18)
C11—C2—C3	118.75 (16)	O1—C12—C13	115.46 (16)
C11—C2—C1	104.35 (15)	C12—C13—H13A	109.5
C3—C2—C1	136.87 (15)	C12—C13—H13B	109.5
C4—C3—C2	124.19 (15)	H13A—C13—H13B	109.5
C4—C3—C8	118.74 (16)	C12—C13—H13C	109.5
C2—C3—C8	117.06 (15)	H13A—C13—H13C	109.5
C5—C4—C3	120.72 (16)	H13B—C13—H13C	109.5
C5—C4—H4	119.6	C19—C14—C15	120.80 (17)
C3—C4—H4	119.6	C19—C14—S1	120.37 (14)
C4—C5—C6	120.68 (17)	C15—C14—S1	118.69 (13)
C4—C5—H5	119.7	C16—C15—C14	120.14 (17)
C6—C5—H5	119.7	C16—C15—H15	119.9
C7—C6—C5	119.72 (18)	C14—C15—H15	119.9
C7—C6—H6	120.1	C15—C16—C17	118.89 (18)
C5—C6—H6	120.1	C15—C16—H16	120.6
C6—C7—C8	121.57 (17)	C17—C16—H16	120.6
C6—C7—H7	119.2	C18—C17—C16	121.34 (18)
C8—C7—H7	119.2	C18—C17—Br1	119.83 (14)
C7—C8—C3	118.50 (17)	C16—C17—Br1	118.84 (15)
C7—C8—C9	121.04 (17)	C17—C18—C19	119.77 (16)
C3—C8—C9	120.45 (18)	C17—C18—H18	120.1
C10—C9—C8	122.03 (18)	C19—C18—H18	120.1
C10—C9—H9	119.0	C14—C19—C18	119.05 (16)
C8—C9—H9	119.0	C14—C19—H19	120.5
C9—C10—C11	116.45 (17)	C18—C19—H19	120.5
			
O2—S1—C1—C12	135.73 (15)	C12—O1—C11—C10	178.95 (18)
C14—S1—C1—C12	−112.55 (16)	C3—C2—C11—O1	178.96 (15)
O2—S1—C1—C2	−48.3 (2)	C1—C2—C11—O1	0.4 (2)
C14—S1—C1—C2	63.40 (19)	C3—C2—C11—C10	−0.8 (3)
C12—C1—C2—C11	0.2 (2)	C1—C2—C11—C10	−179.41 (18)
S1—C1—C2—C11	−176.07 (15)	C9—C10—C11—O1	179.36 (18)
C12—C1—C2—C3	−177.9 (2)	C9—C10—C11—C2	−0.9 (3)
S1—C1—C2—C3	5.8 (3)	C2—C1—C12—O1	−0.8 (2)
C11—C2—C3—C4	−176.38 (17)	S1—C1—C12—O1	176.13 (13)
C1—C2—C3—C4	1.6 (3)	C2—C1—C12—C13	−178.5 (2)
C11—C2—C3—C8	2.4 (3)	S1—C1—C12—C13	−1.6 (3)
C1—C2—C3—C8	−179.6 (2)	C11—O1—C12—C1	1.0 (2)
C2—C3—C4—C5	−179.11 (17)	C11—O1—C12—C13	179.19 (18)
C8—C3—C4—C5	2.1 (3)	O2—S1—C14—C19	−9.52 (16)
C3—C4—C5—C6	0.0 (3)	C1—S1—C14—C19	−123.69 (14)
C4—C5—C6—C7	−1.5 (3)	O2—S1—C14—C15	174.66 (14)
C5—C6—C7—C8	0.9 (3)	C1—S1—C14—C15	60.49 (15)
C6—C7—C8—C3	1.2 (3)	C19—C14—C15—C16	0.4 (3)
C6—C7—C8—C9	−177.87 (19)	S1—C14—C15—C16	176.20 (15)
C4—C3—C8—C7	−2.7 (3)	C14—C15—C16—C17	0.4 (3)
C2—C3—C8—C7	178.44 (17)	C15—C16—C17—C18	−0.5 (3)
C4—C3—C8—C9	176.43 (17)	C15—C16—C17—Br1	179.21 (14)
C2—C3—C8—C9	−2.5 (3)	C16—C17—C18—C19	−0.2 (3)
C7—C8—C9—C10	179.9 (2)	Br1—C17—C18—C19	−179.89 (13)
C3—C8—C9—C10	0.8 (3)	C15—C14—C19—C18	−1.1 (3)
C8—C9—C10—C11	0.9 (3)	S1—C14—C19—C18	−176.81 (13)
C12—O1—C11—C2	−0.9 (2)	C17—C18—C19—C14	1.0 (3)

### Hydrogen-bond geometry (Å, º)

**Table:**

*D*—H···*A*	*D*—H	H···*A*	*D*···*A*	*D*—H···*A*
C19—H19···O2^i^	0.95	2.35	3.221 (2)	152

Symmetry code: (i) −*x*+1, −*y*+1, −*z*.
